# Quantification of Structural Defects Using Pixel Level Spatial Information from Photogrammetry †

**DOI:** 10.3390/s23135878

**Published:** 2023-06-25

**Authors:** Youheng Guo, Xuesong Shen, James Linke, Zihao Wang, Khalegh Barati

**Affiliations:** 1 School of Civil and Environmental Engineering, University of New South Wales, Sydney, NSW 2052, Australia; youheng.guo@student.unsw.edu.au (Y.G.); khalegh.barati@unsw.edu.au (K.B.); 2 Linke & Linke Surveys, 34-36 Byrnes St, Botany, Sydney, NSW 2019, Australia; j.linke@llsurveys.com.au (J.L.); jadynwang@gmail.com (Z.W.)

**Keywords:** crack measurement, crack detection, convolutional neural network, photogrammetry

## Abstract

Aging infrastructure has drawn increased attention globally, as its collapse would be destructive economically and socially. Precise quantification of minor defects is essential for identifying issues before structural failure occurs. Most studies measured the dimension of defects at image level, ignoring the third-dimensional information available from close-range photogrammetry. This paper aims to develop an efficient approach to accurately detecting and quantifying minor defects on complicated infrastructures. Pixel sizes of inspection images are estimated using spatial information generated from three-dimensional (3D) point cloud reconstruction. The key contribution of this research is to obtain the actual pixel size within the grided small sections by relating spatial information. To automate the process, deep learning technology is applied to detect and highlight the cracked area at the pixel level. The adopted convolutional neural network (CNN) achieves an F1 score of 0.613 for minor crack extraction. After that, the actual crack dimension can be derived by multiplying the pixel number with the pixel size. Compared with the traditional approach, defects distributed on a complex structure can be estimated with the proposed approach. A pilot case study was conducted on a concrete footpath with cracks distributed on a selected 1500 mm × 1500 mm concrete road section. Overall, 10 out of 88 images are selected for validation; average errors ranging from 0.26 mm to 0.71 mm were achieved for minor cracks under 5 mm, which demonstrates a promising result of the proposed study.

## 1. Introduction

The problem with aging infrastructure has become one of the most concerning issues globally, especially in developed countries. Infrastructure varies from roads, railways, and buildings to bridges, power plants, and dams. Any potential failure would cause severe impacts to both economic and social life. As most of the crucial infrastructure is made of reinforced concrete, the inspection of these structures is considered a priority. With increasing age, defects such as cracking, spalling, and corrosion could inevitably appear. Wasim, et al. [1] reviewed the durability of geopolymer concrete in the last 20 years. It is reported that 46% of collapsed bridges were already categorized as structurally deficient before the collapse took place, and the collapse rate is estimated as 1/1200 in the New York State of the United States (US) [2]. According to Reagan, et al. [3], the typical designed lifespan for a bridge is 50 years. Structural health monitoring is of great importance to improve infrastructure safety, reduce downtime cost, and prevent catastrophic failure. Currently, 42% of all US bridges are over 50 years old, and 46,154 of the nation’s bridges are considered to be in poor condition [4]. To prevent potential structure collapse, any defects that might lead to major structural failure should be identified at an early stage. Once a certain amount of defects are identified, a decision will be made to repair the defects or even abandon the asset by considering the cost. A comprehensive inspection is indispensable to support the decision-making process.

High cost could be one of the issues hindering the execution of infrastructure inspection. The investment for the maintenance stage is much lower compared to the designing and construction stages. Khan, et al. [5] mentioned that scaffolding, lifting, and protective equipment are needed to conduct remote inspection, which would inevitably increase costs. The cost of crew, traffic control, and involved devices, such as snooper truck and man lift [6,7], could be significant. According to a report from ASCE [4], the investment gap for infrastructure has increased from USD 2.1 to 2.59 trillion every 10 years. It is estimated that the cost for bridge repair is USD 125 billion and the annual budget to improve the bridge condition has increased from USD 14.4 to 22.7 billion. It is noted that the Australian governments and industry have been trying to improve investment in infrastructure gaps since 2015 [8].

Conventional structural inspection is usually completed manually, which requires highly experienced skills and could be risky when accessing dangerous areas (such as working from heights or inside tunnels). Another shortcoming of manual inspection could be inconsistent record keeping. It is hard to record the defect’s exact location on a curved surface such as a power plant or chimney. Moreover, it is time-consuming for one inspector to conduct the examination. If multiple inspectors are involved, errors might appear due to the difference in recognition. Qureshi, et al. [9] also pointed out surface condition rating systems and characteristics to evaluate conditions are not unified.

Recently, some researchers have conducted studies on performing crack assessment using unmanned aerial vehicles (UAVs) [10] and applying deep learning techniques for crack segmentation [11]. Wasim and Djukic [12] reviewed the external corrosion of buried pipelines and up-to-date management methods. Semantic segmentation is one of the emerging technologies that has been adopted in various industrial applications. With the introduction of semantic segmentation for asset inspection, traditional time-consuming and tedious inspection work can be performed automatically. Moreover, as the data is stored in a digital format, a time-based inspection approach can be used to track defect changes over time.

The objective of this paper is to develop an integrated methodology for efficiently measuring minor cracks on concrete structures. Pixel size of an inspection image can be estimated by combining distance information obtained through 3D reconstruction. The first step is separating cracks from the original image with a convolutional neural network and counting the number of pixels for the crack width. Tang, et al. [13] proposed a complete solution including U-Net-based crack segmentation, light and stable backbone extraction, and distribution determination. The pixel sizes are then differentiated by griding the image into small areas and utilizing spatial photogrammetry. Finally, the actual crack dimension is determined by multiplying the pixel number with pixel size. This method has the advantage of being able to estimate defects on complex structures. Although the goal of this method is to monitor the surface condition of large concrete infrastructures accurately and efficiently, access to similar sites is limited. Therefore, to test the feasibility of this method at the initial stage, a pilot case study was conducted on a concrete footpath with cracks ranging from 0.7 to 10 mm. The performance of the proposed method was evaluated using absolute error. Most of the cracks can be identified, and the introduced error was no greater than 0.5 mm.

The structure for the remaining parts of this paper is as follows. In Section 2, previous work on CNN-based semantic segmentation, image-based crack quantification, and 3D reconstruction is reviewed. In Section 3, an integrated methodology is presented to conduct crack measurement utilizing semantic segmentation and computer vision, including data capturing, crack detection, and crack measurement. In Section 4, a case study on the cracks on a concrete footpath is performed to prove the applicability of obtaining grid pixel size. In Section 5, the gaps and limitations of the study are discussed. Finally, a conclusion is drawn in Section 6.

## 2. Related Work

As the traditional inspection is conducted by humans [14], the number of cracks may be underestimated due to limited access and unavoidable human error. To overcome these drawbacks and minimize the budget for infrastructure inspections, a series of advancements have been made to optimize the non-contact workflow from data capture and defect detection to defect quantification. Initially, cameras and light detection and ranging (LiDAR) sensors were used to capture structural defects. However, due to the balance between capability, compatibility, and cost, cameras are considered the best option for inspecting minor defects such as cracks. Therefore, two-dimensional red, green and blue (RGB) images will be the primary data source in this study. Moreover, the detection process was automated using machine learning techniques.

### 2.1. Defect Detection Based on Machine Learning

Since the introduction of AlexNet [15], CNN has been widely applied in many industries. In recent years, researchers have been introducing deep learning-based techniques for crack detection.

Manual inspection has been widely conducted in recent years due to the crucial role of experience in this field. However, the introduction of artificial intelligence (AI) has the potential to reduce the burden on inspectors by limiting the area of interest. In particular, AI has rapidly developed in the realm of two-dimensional imaging. Semantic segmentation algorithms, which are typically based on convolutional neural networks, have become a mature technology in computer science. For instance, Krizhevsky, Sutskever and Hinton [15] trained a deep CNN network with 60 million parameters, achieving a significant score in the ImageNet contest. Zeiler and Fergus [16] further explained the workings of the AlexNet model and developed a superior architecture. Szegedy, et al. [17] proposed an Inception network that optimized the utilization of computing resources. Ronneberger, et al. [18] also presented an efficient strategy for maximizing the use of annotated samples. Furthermore, Szegedy, Vanhoucke, Ioffe, Shlens and Wojna [19] established principles for designing high-performance networks with low computational costs.

Based on CNN, many researchers have developed different structures for different purposes. Defect detection is one of the semantic segmentation applications in the Architecture, Engineering, and Construction (AEC) industry. Semantic segmentation-driven crack detection is more objective and reliable compared to the traditional manual inspection [20]. Oliveira and Correia [21] proposed an automatic system for crack detection and characterization, and the algorithm could detect multiple cracks from 56 images in about two minutes. Chen, et al. [22] suggested a simple and improved structure of convolutional neural networks achieving high accuracy. The authors believed that a large convolution and pooling methodology with fewer network layers could be utilized to obtain a better result for simple crack identification. By setting the learning rate to 0.01, Li and Zhao [23] developed an algorithm with high accuracy based on CNN structure and AlexNet. Liu, et al. [24] adopted U-Net for high efficiency and robustness. Dung [25] proposed a crack detection method based on FCN for semantic segmentation on concrete crack images. Bang, et al. [26] proposed a deep convolutional encoder-decoder network-based method to identify road cracks from black-box images. The automated crack identification and visualization algorithm used by Jang, et al. [27] is enabled by transfer learning from GoogleNet. Qu, et al. [28] applied LeNet-5 to classify the cracks and optimized VGG16 to extract concrete crack characteristics. Chow, et al. [29] provided an artificial intelligence-based inspection workflow for anomaly detection and reduced the search space of defects up to 80% for minor defect regions. Dais, et al. [30] firstly applied deep learning techniques on masonry images with pixel-level segmentation. Miao and Srimahachota [31] combined a trained CNN and an image processing method to detect and quantify cracks in a semi-automatic way. Fu, Meng, Li and Wang [6] proposed an algorithm based on Dense-DeepLabv3+ network to segment bridge crack images. Ali, et al. [32] reviewed the applications of CNN on civil crack detection. Wang and Su [33] suggested the SegCrack model, including a hierarchically structured transformer encoder to output features and a top-down pathway with lateral connections to up-sample and fuse features. Moreover, Xu, et al. [34,35] applied deep neural networks for 3D object detection over as-built reconstruction and automated scan-to-BIM. Although much research has been conducted on crack detection, the labeled area is still not accurate enough for minor cracks.

### 2.2. Defect Measurement with Image Processing and Photogrammetry

To measure the actual dimensions of cracks, researchers have performed experiments to extract the information from images. However, lens distortion and projective transformation can result in inaccuracies in the measurements. Therefore, reconstructing cracks in three-dimensional space is one of the most reliable ways for quantifying cracks.

Cho, et al. [36] presented a five-step method to improve the accuracy and consistency of measuring crack width. Albareda-Valls, et al. [37] tested an image post-processing method to quantify cracks on concrete elements. Vashpanov, et al. [38] developed a method to determine crack dimensions based the pixel intensity distribution of images and achieved an accuracy of less than ±15%. Liu, Nie, Fan and Liu [10] concluded that the assessment of cracks can be concluded as filtering noise and extracting parameters. Bang, et al. [39] used structured lights and depth cameras to quantify structural damage. Wang, et al. [40] proposed a key point method for crack characterization and established a crack model based on anchor points. Shi, et al. [41] reconstructed 3D images based on structured illumination. Fan, et al. [42] proposed a method to measure crack dimensions by extracting crack skeletons from images. Parente, et al. [43] proposed a machine learning-based method that only requires a single image for training and provides accurate outputs.

The issue with the aforementioned approaches is that the quantification process is only based on a two-dimensional image, which neglects the information of the third dimension. The influence of lens distortion and projective transformation should also be considered. One of the most recent solutions is adopting computer vision and close-range photogrammetry to reconstruct the defects in three dimensions.

Jahanshahi and Masri [44] proposed a contactless quantification method for cracks based on computer vision and image processing. Liu, et al. [45] proposed a solution to locate cracks by combining 2D image and 3D scene reconstruction. Yang, et al. [46] proposed a damage-indexing method that integrates image-based crack measurement and crack quantification methods. Kalfarisi, et al. [47] used a 3D reality mesh for quantitative assessment. Wu, et al. [48] combined UAV-taken photos and Mask-RCNN to construct a 3D water tower model with highlighted cracks. Building upon previous results, Liu, Nie, Fan and Liu [10] presented a new crack assessment approach using UAVs and 3D scene reconstruction to inspect bridge piers. The authors also presented a method of projecting cracks onto a 3D mesh surface, which eliminates distortion on non-flat surfaces. Chaiyasarn, et al. [49] detected a large range of cracks on a 3D mesh model by creating an artificial camera position. Shokri, et al. [50] proposed a planar and matching method for 3D crack reconstruction with higher accuracy and faster speed. Zhao, et al. [51] presented a system of camera and laser rangefinder to measure the width of cracks from different angles and distances. Woo, et al. [52] used relative objects in the image to rectify the location of cracks without GPS information, which can potentially improve the accuracy of the measurement results.

Although much research has been conducted to measure the dimensions of cracks from 2D images, some limitations remain. The distance between the target and camera is typically fixed or manually measured, which is time-consuming, especially when multiple images are needed for photogrammetry. Additionally, traditional crack quantification can only be performed on simple flat surfaces.

## 3. Research Methodology

**Figure 1:**
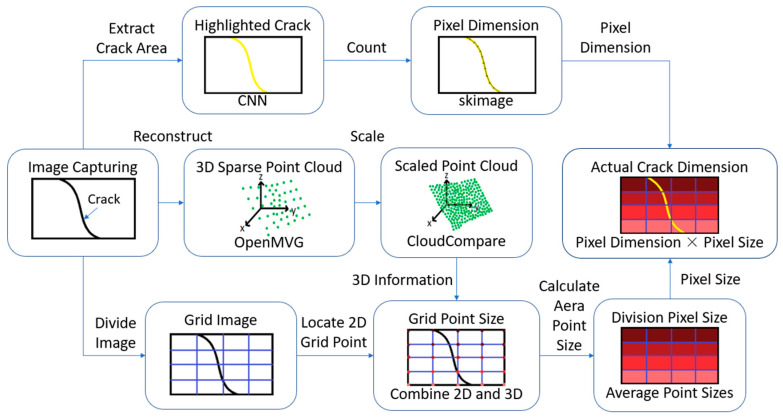
The workflow of crack quantification.

In this paper, a method based on 3D reconstruction and semantic segmentation will be adopted to acquire the pixel size information as shown in Figure 1. To measure the actual dimension of cracks from an image, distance information is needed. The most direct way is to measure the distance while taking photos. However, this process could be time-consuming and inaccurate. The obtained image in this study will be processed in three directions: automatic pixel dimension extraction, three-dimensional reconstruction, and grid point location.

### 3.1. Pixel Level Semantic Segmentation for Defect

To derive the crack pixel dimension from obtained images, the first step is to separate cracks from the background automatically. With the development of deep learning technology, CNN-based semantic segmentation technology is utilized to automatically detect the cracked area. This section will present a practical workflow to implement the two-dimensional artificial intelligence for crack detection.

Referring to Simonyan and Zisserman [53], VGG16 is adopted as the encoder. It initially has 16 weight layers, and each layer consists of Maxpool and Convolution + Batch Norm + ReLU. After testing, it was found that adding Batch Norm between Convolution and ReLU could improve performance. The proposed convolutional neural network model is adjusted for concrete cracks [54].

The input image was cut into 448 × 448 and fed into the algorithm as a [448 × 448 × 3] matrix. The decoder is designed for crack detection, each layer consists of Bilinear Interpolate and Convolution Kernel Size 3 + ReLU. The dimensions of each layer are listed below (see Table 1).

The final layer consists of Convolution (kernel size 3) + ReLU, Convolution Kernel Size 1, and Log SoftMax. The dimension of the output image is [448 × 448 × 1], and the value of each pixel is either 0 or 1, representing crack or non-crack. However, since the number of crack and non-crack images is not equal, the traditional Binary Cross Entry (BCE) loss tends to regard the image as not having cracks. To overcome this issue, a focal loss method based on the structure proposed by Lin, et al. [55] is applied for classification. Backpropagation is then performed to adjust the parameters. The VGG16 + Focal Loss model was trained on a smaller dataset with fewer epochs. Although ResNet is commonly used for crack detection, U-Net [27], which is one of the recent developments based on ResNet, will also be compared with VGG16 + Focal Loss and VGG16 + BCE Loss (see Table 2). The overall performance of VGG16 + Focal Loss, with an F1 Score of 0.613, is better than the other two models.

**Figure 2:**
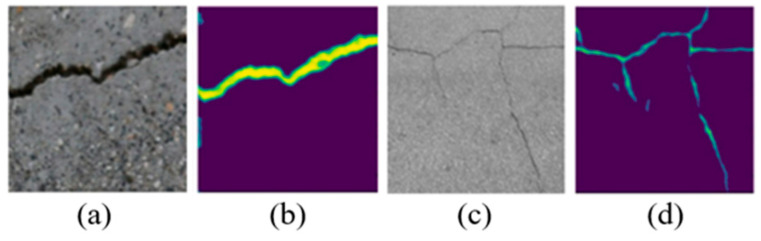
The results of different models for obvious and minor cracks: (**a**) original image of obvious cracks, (**b**) VGG16 + Focal Loss with more training data sets for obvious cracks, (**c**) original image of minor cracks, and (**d**) VGG16 + Focal Loss with more training data sets for minor cracks.

The results of annotated images for some obvious cracks and thin cracks are presented in Figure 2.

Next, the pixel dimensions of the cracks can be determined. The number of pixels within the highlighted area will be counted to represent the crack dimension. Crack measuring is based on the labelled crack image created in the previous section. Since the pixels in the crack area are set to 1 and the rest of the pixels are labelled as 0 for non-crack, the “skimage” package was applied to draw the boundary of the cracked area. Then, a skeleton of the crack will be created at the center of two longitudinal lines. After that, a line of width will be created perpendicularly to the skeleton, as proposed by Cho, Yoon and Jung [39].

To obtain the real dimension of the cracks, multiplying the pixel dimension with the scale factor will be needed. In this scenario, pixel size as the third-dimension information is the key issue. In the follow-up sections, scale factors will be obtained from photogrammetry-enabled 3D reconstruction.

### 3.2. Pixel Size Quantification Using Spatial Information from Photogrammetry

For most of the crack measurement process, pixel sizes are normally regarded as the same. Therefore, the main contribution for this research is to differentiate pixel sizes in different areas. Ideally, this method will make it possible to measure the defects on images taken from different angles. The workflow will be presented in the following sections.

To overcome the limitation of traditional inspection methods, photogrammetry algorithms are selected to obtain the spatial information by reconstructing the defects in 3D. As the exported point cloud model is not scaled, control points or references will be needed to convert the model to actual size. Moreover, the connecting information between 2D and 3D tie points will also be of great importance for the next procedures.

Grids are applied to divide the image into multiple small sections with different pixel sizes. The number of grids depends on the desired accuracy for crack measurement. In this paper, each image is divided into 8 × 8 areas to differentiate pixel sizes. Grid points are used to assist calculations within different areas.

**Figure 3:**
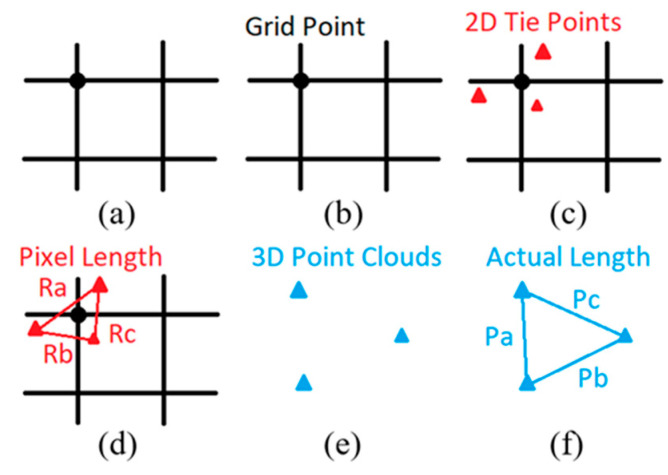
Flowchart of calculating grid pixel size: (**a**) grid, (**b**) corner pixel, (**c**) nearest three tie points on image, (**d**) pixel perimeter, (**e**) corresponded three 3D tie points, (**f**) actual perimeter.

The flowchart of calculating grid pixel size is presented in Figure 3. To obtain the actual size of corner grid pixels, the first step is to locate the 2D pixel in scaled 3D point clouds. However, the corner pixel at the grid point in the image does not usually have a corresponding tie point because the tie point cloud is relatively sparse. Therefore, instead of using the exact grid point pixel, the nearest three 2D tie points around the corner point will be adopted.

This paper adopts the perimeter method instead of the area method to calculate the grid pixel size, as the calculation can overcome the scenario when three points are located on the same line and computation speed is faster. The grid pixel size can be calculated using Equation (1). The distances between three points can be labelled as Pa, Pb, and Pc (pixels). Then, using the exported corresponding information, relate 2D coordinates to 3D coordinates in point clouds. The distances between three points can be labelled as Ra, Rb, and Rc (mm).

(1) $$Grid\;Pixel\;Size=\frac{Ra+Rb+Rc}{Pa+Pb+Pc}mm/Pixel$$

Knowing the pixel size at the grid point, the next step is to derive the area pixel size. By averaging the values of four grid points at corners, area pixel sizes within that area can be obtained. For better visualization, pixel sizes are represented by different color brightness (the larger pixel size has a brighter color). Since pixel dimension and pixel size are known, the actual dimension of the cracks in different sections can then be calculated by multiplying the two values. To validate the accuracy, the exported results will be compared with the gauge measured value and dimensions measured from the point cloud.

## 4. Experiments and Validations

**Figure 4:**
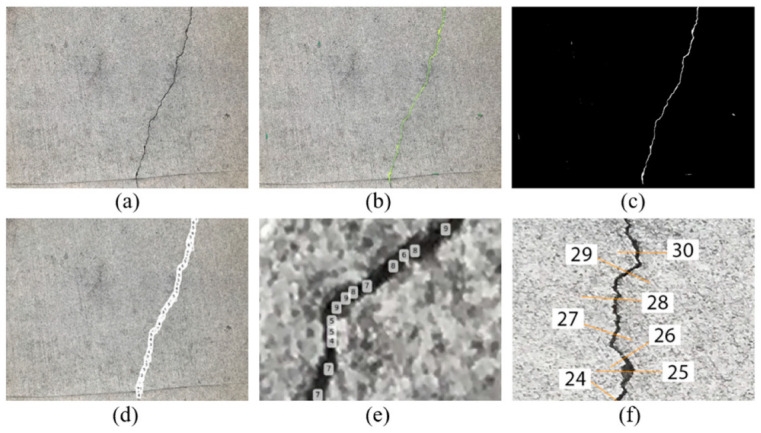
The process of measuring crack pixel dimension: (**a**) raw image of the crack; (**b**) labelled crack; (**c**) and crack mask; (**d**) automatically counted crack width in pixel; (**e**) partially zoomed in crack; (**f**) part of labelled measurement spots on the target crack.

**Figure 5:**
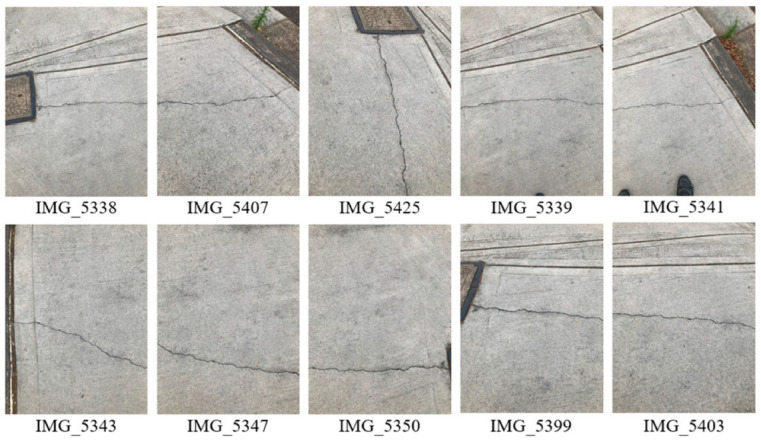
Ten images shot from different angles.

To validate the feasibility of the proposed methodology, a pilot case study was performed over a small-scale site which is a concrete footpath (approximately 1500 mm × 1500 mm) with a long crack distributed on the flat surface. Instead of a flying UAV, an Apple iPhone 7 was used to capture images for testing purposes. The average crack width is about 3.15 mm. Approximately 88 images with a resolution of 4032 × 3024 pixels were taken of the crack. The raw images, processed images, and part of the measured spots on the crack are labelled in Figure 4. Ten images took from different angles were selected to validate the proposed methodology (see Figure 5).

### 4.1. Crack Width in Pixel

As mentioned in the methodology, the proposed semantic segmentation algorithm was applied to the images to highlight the cracked area with yellow. The labelled crack on the original image and mask are presented in Figure 4b and Figure 4c, respectively. The pixel dimension is automatically labelled, as can be seen from Figure 4d,e.

### 4.2. Spatial Information

**Figure 6:**
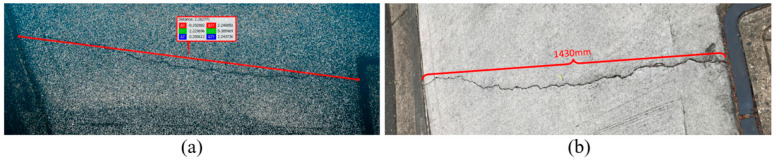
The process of defining 3D scale factor: (**a**) the referenced length of the crack; (**b**) the referenced length of the crack.

The next step is to obtain the spatial information. photogrammetry is applied to perform 3D reconstruction based on the captured images. A sparse point cloud (see Figure 6a) is created in this case study as the accuracy is good enough.

As the model exported from COLMAP is in an arbitrary unit system, a referenced length or ground control point (GCP) is needed to scale the model to actual dimension. In this case study, the arbitrary length of the crack is measured as 2.26 from the point cloud as can be seen in Figure 6a and the real length is measured as 1430 mm (see Figure 6b). By dividing the real length by the arbitrary value, the 3D point cloud scaled factor can be derived as 632, which will be applied to calculate the width of each crack.

The information generated and exported from photogrammetry is crucial as it contains the corresponding information between 2D tie points on the image and 3D point clouds. By selecting a pixel on the image, the spatial information can be derived through the exported data file.

### 4.3. Grided Image

The third stage is to calculate various pixel sizes along the crack. To achieve this goal, the image is divided into small sections. Theoretically, the smaller the division, the more accurate the result will be. In this case study, the image is segmented into an 8 × 8 grid as a preliminary test. As the original size of the image is 4032 × 3024, it is divided into 64 pieces of 504 × 378 elements.

**Figure 7:**
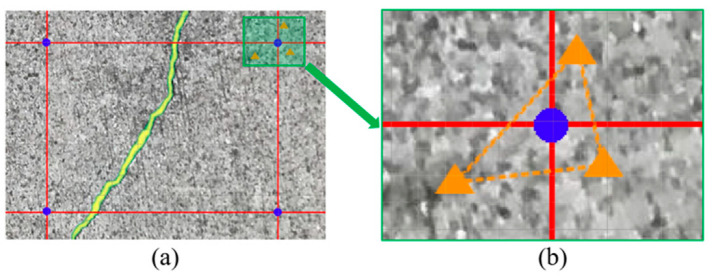
Calculate the pixel size of corner grid: (**a**) corner point; (**b**) nearest three tie points.

The intersections are labelled with blue dots in Figure 7a. The blue dots are corner points of the rectangular area that represent the pixel sizes within the area. The process is described as follows.

The first step is obtaining the size of the corner point. As displayed in the figure, the corner point has a specific coordinate on the image. The nearest three tie points are found and labelled as orange triangles (see Figure 7b). The perimeter of the formed triangular area can be calculated in pixel unit. Then, the 3D coordinates of the three tie points can be found by linking information generated from photogrammetry. From there, the 3D perimeter in arbitrary units can be calculated, and the pixel size within that area can be calculated by dividing the 3D perimeter with the 2D pixel perimeter. Therefore, the blue dot size can be derived as the corner point size is subjected to the pixel sizes within the triangular area.

**Figure 8:**
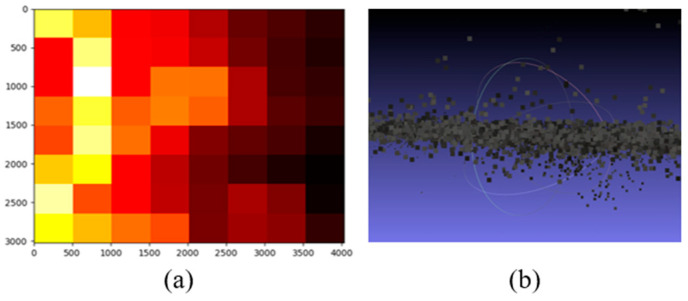
(**a**) Heat map on pixel size of an image; (**b**) noise of the created point cloud surface.

Since the pixel size at the corner points is known, the pixel size within the rectangular area can be calculated by averaging the sizes of the four corner points. After that, a chart showing different pixel sizes in different areas can be mapped as seen in Figure 8a. Since further pixels correspond to larger real dimensions in the image, they are represented by lighter colors on the map. However, the transition is not 100% correct. One of the main issues is that the noise generated along the point cloud can lead to inaccurate results as seen in Figure 8b.

### 4.4. Derivation of the Actual Size

**Figure 9:**
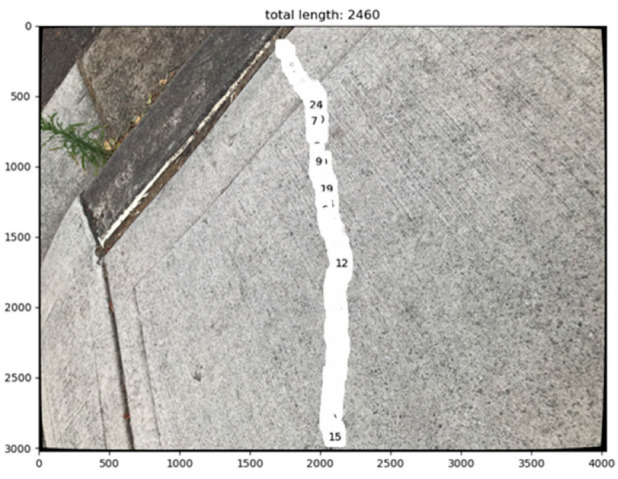
Automatically counted pixel number.

Based on the automatically counted pixel width and pixel size map as displayed in Figure 9. The actual dimension can be calculated in Equation (2).

(2) Calculated Width=3D Scale Factor×Pixel Width×Pixel Size

### 4.5. Validation

Statistical results will be created by comparing the calculated results with the gauge measured results.

According to the Australian Standard 2870 [56], damage levels for different crack widths are presented as below (see Table 3). Most of the cracks in this paper can be regarded as wide cracks.

**Figure 10:**
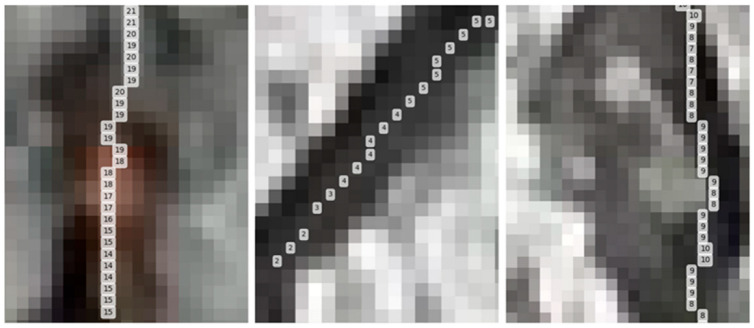
Some obvious errors caused by the detection algorithm.

IMG_5403 is selected to present the results of proposed crack measurement. The distribution of errors in this image is typical among the ten images. As can be seen from Table 4, most errors are around 1 mm. However, several errors are larger than 1.5 mm (e.g., 1.84 mm, 2.51 mm, and 3.01 mm). Several factors could cause the large errors, such as an inaccurate pixel scale factor. One of the most common issues is that the detection algorithm might export the wrong pixel number, as can be seen from Figure 10.

As the outliers could affect the accuracy, when calculating the average error, two sets of data are presented in Table 5. The first row of errors is calculated including errors less than 1.5 mm and the second row are calculated with all data. It can be found that the proposed workflow of crack measurement could lead to an error of 0.48 mm for 3.32 mm mean widths.

## 5. Discussions and Limitations

In this research, an innovative approach is proposed to determine the dimension of minor defects. With the application of CNN and photogrammetry, the shape of a crack can be automatically extracted, and the pixel size information can be determined. Compared to the conventional methods, the new method makes it possible to accurately quantify the defect from image.

Although the accuracy of the proposed methodology has been validated in the previous section, some issues were found that might affect the result, including point cloud noise, cast of shadows, irregular shape, and shooting angle.

Further research will be focusing on estimating individual pixel sizes while saving computational resources and increasing processing speed. Moreover, by deploying UAV, large-scale experiments will be performed on more complicated infrastructures, such as bridges, dams, and cooling towers to prove the feasibility of the proposed pixel-level method.

## 6. Conclusions

Many research efforts have been devoted to quantifying the dimension of cracks from images for aging infrastructures, such as bridges, roads, dams, and tunnels. Depth information derived from the close-range photogrammetry is omitted in most studies. This paper discussed the relationship between the real dimension and corresponding pixels on a 2D image. It provides an efficient solution to automatically detect and accurately measure the dimensions of minor cracks. The pixel size is obtained by leveraging spatial data exported from point cloud reconstruction. A case study was performed on a concrete footpath with cracks distributed on the surface in Sydney, and the results proved the feasibility of the proposed methodology.

Some improvements can be made in future research, such as enhancing the accuracy of the semantic segmentation algorithm, denoising the surface, and reducing the size of the grided section. The integration of LiDAR with images could provide an alternative approach to simplify and speed up the process of pixel size determination. Ideally, the proposed technology can be applied to provide accurate defect quantification and realize real-time asset inspection, ultimately improving the safety of public infrastructure.

## Figures and Tables

**Table 1:** The dimensions of each layer in the encoder.

No. of Layer in Encoder	Dimension	No. of Layer in Decoder	Dimension
1	224 × 224 × 64	5	28 × 28 × 256
2	112 × 112 × 128	4	56 × 56 × 256
3	56 × 56 × 256	3	112 × 112 × 64
4	28 × 28 × 512	2	224 × 224 × 32
5	14 × 14 × 512	1	448 × 448 × 32

**Table 2:** The comparison between three models.

	Baseline U-Net	VGG16 + BCE Loss	VGG16 + Focal Loss
Average Precision	0.616	0.432	0.566
Average Recall	0.582	0.603	0.670
F1 Score	0.598	0.503	0.613

**Table 3:** Categories for damage on slab.

Description of Typical Damage	Approximate Crack Width Limit	Change in Offset in 3 m Straight Edge	Damage Category
Hairline crack	<0.3 mm	<8 mm	0
Fine crack	<1.0 mm	<10 mm	1
Distinct crack	<2.0 mm	<15 mm	2
Wide crack	2–4 mm	15–25 mm	3
Gaps in slab	4–10 mm	>25 mm	4

**Table 4:** Validation result from IMG_5403.

Crack No.	Ground Truth (mm)	Pixel Scale Factor	Automatically Counted Pixel Number	Calculated Width (mm)	Error (mm)	Absolute Error (mm)
30	2.50	0.000329	12	2.50	0.00	0.00
34	2.50	0.000392	10	2.48	−0.02	0.02
33	1.40	0.000332	7	1.47	0.07	0.07
24	3.00	0.000384	13	3.15	0.15	0.15
25	7.00	0.000337	32	6.82	−0.18	0.18
28	3.00	0.000329	13	2.70	−0.30	0.30
29	3.00	0.000329	13	2.70	−0.30	0.30
26	3.50	0.000337	18	3.83	0.33	0.33
32	2.50	0.000332	10	2.10	−0.40	0.40
23	2.00	0.000332	7	1.47	−0.53	0.53
27	2.50	0.000337	15	3.19	0.69	0.69
19	3.00	0.000395	9	2.25	−0.75	0.75
20	4.00	0.000395	11	2.75	−1.25	1.25
35	2.50	0.000392	5	1.24	−1.26	1.26
16	3.00	0.000511	14	4.52	1.52	1.52
14	3.00	0.000511	15	4.84	1.84	1.84
18	2.50	0.000528	15	5.01	2.51	2.51
17	3.00	0.000528	18	6.01	3.01	3.01

**Table 5:** Average error for each image (mm).

	IMG 5338	IMG 5339	IMG 5341	IMG 5343	IMG 5347	IMG 5350	IMG 5399	IMG 5403	IMG 5407	IMG 5425
Average errors for cracks less than 1.5 mm	0.26	0.49	0.53	0.45	0.53	0.42	0.36	0.45	0.71	0.60
Average errors for all cracks	0.90	0.74	0.57	0.56	0.72	0.52	0.66	0.84	0.80	1.01
Average widths	3.86	3.35	2.72	2.51	3.54	4.04	3.63	2.99	2.28	4.27

## Data Availability

Not applicable.
